# Lipoprotein Signal Peptide as Adjuvants: Leveraging Lipobox-Driven TLR2 Activation in Modern Vaccine Design

**DOI:** 10.3390/vaccines13010036

**Published:** 2025-01-02

**Authors:** Muhammad Umar, Haroon Afzal, Asad Murtaza, Li-Ting Cheng

**Affiliations:** 1 Graduate Institute of Animal Vaccine Technology, College of Veterinary Medicine, National Pingtung University of Science and Technology, Pingtung 91201, Taiwan; 2 International Program in Animal Vaccine Technology, International College, National Pingtung University of Science and Technology, Pingtung 91201, Taiwan; 3 Faculty of Biosciences, Fisheries and Economics, Norwegian College of Fishery Science, UiT—The Arctic University of Norway, P.O. Box 6050 Tromsø, Norway

**Keywords:** lipobox, SP, TLR2, adjuvant, bacterial lipoprotein

## Abstract

Toll-like receptor 2 (TLR2) signaling is a pivotal component of immune system activation, and it is closely linked to the lipidation of bacterial proteins. This lipidation is guided by bacterial signal peptides (SPs), which ensure the precise targeting and membrane anchoring of these proteins. The lipidation process is essential for TLR2 recognition and the activation of robust immune responses, positioning lipidated bacterial proteins as potent immunomodulators and adjuvants for vaccines against bacterial-, viral-, and cancer-related antigens. The structural diversity and cleavage pathways of bacterial SPs are critical in determining lipidation efficiency and protein localization, influencing their immunogenic potential. Recent advances in bioinformatics have significantly improved the prediction of SP structures and cleavage sites, facilitating the rational design of recombinant lipoproteins optimized for immune activation. Moreover, the use of SP-containing lipobox motifs, as adjuvants to lipidate heterologous proteins, has expanded the potential of vaccines targeting a broad range of pathogens. However, challenges persist in expressing lipidated proteins, particularly within heterologous systems. These challenges can be addressed by optimizing expression systems, such as engineering *E. coli* strains for enhanced lipidation. Thus, lipoprotein signal peptides (SPs) demonstrate remarkable versatility as adjuvants in vaccine development, diagnostics, and immune therapeutics, highlighting their essential role in advancing immune-based strategies to combat diverse pathogens.

## 1. Introduction

Vaccination is recognized as one of the most efficient and economically viable breakthroughs in medicine, saving millions of lives across the globe. Diseases like smallpox, polio, and measles have been effectively eradicated via immunization [1,2,3,4]. Despite the success of vaccination against some diseases, several infectious diseases do not yet have vaccines [5]. Historically, most vaccine formulations contain live attenuated or inactivated pathogens. However, issues such as reversion to virulence, undesired host reaction, and the difficult cultivation of pathogens are associated with the use of traditional vaccine preparation methods. This unviability resulted in the advent of subunit vaccines, which are largely safe [6]. However, subunit vaccines are poorly immunogenic by themselves and need an adjuvant to trigger the host immune system [7]. Since 1970, several efforts have been made to develop effective adjuvants but only a few (alum, MF59, AF03, AS02, and AS04) have been licensed for clinical application [8]. Adjuvants must meet stringent criteria to obtain approval for a clinical application, for example, being non-toxic, producing robust humoral/T-cell response, ensuring long-term immunity, and not triggering an autoimmune or allergic response [9]. Recent advances in vaccine designs have been greatly influenced by the discovery of signaling pathways involved in the innate and adaptive immune responses. In this regard, toll-like receptors (TLRs) are widely recognized as key players in bridging innate and adaptive immune response [10]. Direct targeting of TLRs employing the subunit vaccine design alongside built-in TLR agonists has become a popular strategy. Flagellin (TLR5 agonist) and bacterial lipoproteins (TLR2 agonist), and CpG (TLR9) are being widely used as adjuvants in subunit vaccines [11,12,13,14,15,16].

**Table 1:** Signal peptide as adjuvant in various antigen models.

Pathogen/Condition	Antigen	SP (SP)	Immune Response	References
*Dengue Virus*	E3 protein	Neisseria meningitidis Ag473	Higher IgG and virus-neutralizing antibodies in lipidated E3	[17]
*Clostridium difficile*	TcdA Receptor-Binding Domains (RBD)	Ag473	A 10-fold increase in potency; 90–100% protection against CDI	[18]
*Staphylococcus aureus*	FLIPr (Formyl Peptide Receptor-Like 1 Inhibitor Protein)	Ag473	Boosted mucosal and systemic immunity	[19]
*Zika Virus*	Envelope Protein Domain III (rZE3)	Ag473	Higher neutralizing antibodies with prolonged protection	[20]
*Haemophilus influenzae*	P6 and OMP26 proteins	P4 SP	Higher antibody titers and cytokine responses	[21]
*Human Papillomavirus (HPV)*	E7 protein	Ag473	Increased anti-E7 antibodies with Th1-biased cytokine release	[22]
*Streptococcus pneumoniae*	DacB and PnrA	Signal sequence from *B. burgdorferi*	Increased IgG2/IgG1 subclass ratios related to Th1-type	[23]

## 2. Bacterial Lipoproteins: Driving Immune Modulation and Vaccine Advancement

**Figure 1:**
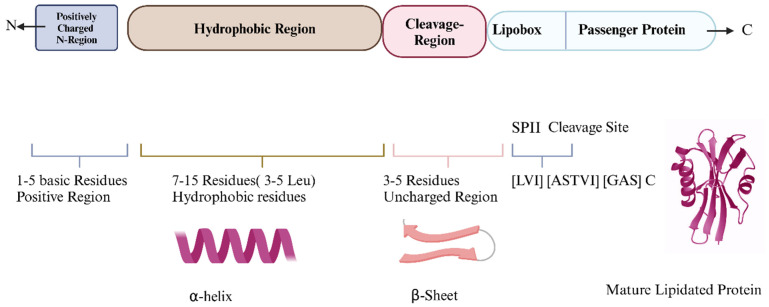
Signal peptides: structure and function in protein targeting (Created in https://BioRender.com—accessed on 15 November 2024).

Bacterial lipoproteins are essential membrane proteins involved in bacterial physiology, structural integrity, signaling, and host–pathogen interactions. Ubiquitous among Gram-positive and Gram-negative bacteria, these lipoproteins are characterized by a polypeptide chain linked with lipid moiety. The lipid group is attached covalently with the N-terminal cysteine residue of the polypeptide chain, a process known as lipidation. This lipidation allows the bacterial lipoproteins to anchor with the membrane and facilitate further biological activities. Naturally, the biogenesis of the bacterial lipoproteins initiates within the cytoplasm as a precursor coupled with enzymatic processes and equipped with an SP. SPs are mandatory for the post-translational modification as well as the translocation of the nascent protein [24,25]. First postulated by Blobel and Heijne, the SP (typically 16–30 amino acids) is characterized by positively charged amino acids (commonly lysine/arginine) at their N-terminal, a central hydrophobic region (7–15 amino acids), and a conserved lipobox motif (Leu-(Ala/Ser)-(Gly/Ala)-Cys) at the C-terminal [26]. The cysteine residues of the lipo-box motif are notable as it becomes the site of lipidation during post-translational modification, necessary for biological roles including immune activation [27]. Figure 1 illustrates the structure of the SP and lipo-box motif.

Signal peptides guide protein targeting in the following three regions: a positively charged N-terminal, a hydrophobic core for membrane embedding, and a C-terminal cleavage site with a lipobox for lipid modification. Cleavage by SPase produces a lipidated protein.

These post-translationally modified bacterial lipoproteins are known for their interaction with the host immune system employing pathogen recognition receptors (PPRs) such as toll-like receptor 2 (TLR2). The recognition of the bacterial lipoproteins is mediated by the lipidated N-terminal cysteine residues, which interact with the TLR2 while forming heterodimers with either TLR1 or TLR6. Tri-acylated lipoproteins are recognized by the TLR1-TLR2 heterodimer, while diacylated lipoproteins interact with TLR6-TLR2 heterodimers. Upon recognition by the toll-like receptor, it triggers the signaling cascade that results in the activation of the nuclear factor kappa light chain enhancer of activated B cells (NF-κB), mitogen-activated protein kinases (MAPK), and interferon regulatory factors (IRFs) [28]. This signaling provides the basis for innate immune response and results in the production of pro-inflammatory cytokines (TNF-α, IL-1β, IL-6, IL-8, IL-12, IL-18) and further bridges the adaptive immune response as well. This immune fostering mechanism positions bacterial lipoproteins as potential vaccines and therapeutic candidates [29].

Beyond the natural biogenesis of bacterial lipoproteins, synthetic/chemical lipidation has been introduced aimed at mirroring the natural process of lipidation that occurs in bacterial lipoproteins. During synthetic lipidation, specific lipid moieties (Pam2CSK4 or Pam3CSK4) are attached covalently with proteins or peptides at the cysteine residues of the lipobox [30]. Synthetic lipidation enables the manipulation of protein localization and function by mimicking natural lipid modifications such as prenylation (attachment of isoprenoid lipids such as farnesyl and geranylgeranyl groups to cysteine residues), fatty acylation (addition of fatty acids such as palmitate or myristate to cysteine residues or the N-terminus), and GPI anchoring (attachment of glycosylphosphatidylinositol at the C-terminus) [31]. Because of the inherent hydrophobicity of lipids, synthetic procedures require precise strategies to ensure optimum yield, selectivity, and functionality of lipid–protein conjugates. Synthetic lipidation allows for detailed structure–function study and has applications in drug delivery systems by binding antibodies or therapeutic proteins to lipid bilayer carriers. This chemical lipidation not only improves the immunogenicity of proteins or peptides, but also enhances their biological function such as pharmacokinetic and pharmacodynamic properties [32]. These lipidated proteins or peptides are recognized by the pathogen recognition receptors as danger signals (TLR2 and TLR1/TLR6). Thus, bacterial proteins coupled with lipidation are promising vaccine candidates [33].

Bacterial lipoproteins are also attributed to the pathogenicity of many bacteria and are often involved in virulence and immunomodulatory processes. For instance, *Mycobacterium tuberculosis* lipoprotein LprG binds the triacylated glycolipid agonists of TLR2 and enhances their recognition by TLR2 [34]. Similarly, lipoprotein (PsaA) from *Streptococcus pneumoniae* is responsible for immune modulation. Using the surface-lipoprotein deficient mutant strain of *S. pneumoniae* results in the reduced activation of NF-κB and TNF-α release. This testifies that lipoproteins are essential for the TLR2-mediated inflammatory response [35].

Based on the aforementioned mechanism, some vaccine products that have been already commercialized contain bacterial lipoproteins. The outer surface protein A (OspA) from *Borrelia burgdorferi*, causative agent for Lyme disease, was the first studied bacterial lipoprotein. Toll-like receptor 2 (TLR2) plays a crucial role in the immune response to the outer-surface lipoprotein A (OspA) of *B. burgdorferi*, the causative agent of Lyme disease, by recognizing tripalmitoyl-S-glyceryl-cysteine (Pam3Cys)-modified lipoproteins. The Pam3Cys modification enhances the adjuvant activity of OspA, leading to increased humoral responses, as evidenced by reduced immunoglobulin G (IgG) levels in TLR2-deficient mice compared to wild-type mice. However, IgG1 production remained similar in both strains, and protective immunity levels were comparable, indicating that the lipid modification’s adjuvant properties can operate independently of TLR2 signaling [36]. Recombinant OspA, produced in *Escherichia coli* and purified through detergent extraction and ion-exchange chromatography, elicited strong IgG responses and provided protection against spirochetal challenges even without adjuvants. In contrast, non-lipidated OspA showed no immunogenicity [37]. Additionally, a study identified low responders among vaccinated individuals who exhibited diminished cytokine production and TLR1 expression upon OspA stimulation, while TLR2 levels were normal. Mice lacking TLR1 or TLR2 produced low antibody titers against OspA, with TLR2-deficient macrophages being unresponsive to both OspA and peptidoglycan. These findings suggest that defects in the TLR1/2 signaling pathway may contribute to human hypo-responsiveness to the OspA vaccination [38].

Later, Trumenba (bivalent rLP2086), licensed for preventing *Neisseria meningitidis* serogroup B (NmB) disease in individuals aged 10–25 years, showcases the critical role of lipidation in its vaccine efficacy. Comprising two recombinant factor H-binding protein (fHbp) variants, rLP2086-A05 and rLP2086-B01, the vaccine induces robust bactericidal antibodies against diverse NmB isolates due to its structural components, the polypeptide domain, essential for antigenicity, and the N-terminal lipid moiety, which enhances stability and immune response [39,40,41]. The lipoproteins self-associate into micelles driven by lipid hydrophobicity, facilitating their structural integrity in the absence of bacterial cell walls. Notably, the lipid moieties confer toll-like receptor (TLR) 2 agonist activity; their removal diminishes this immune activation, underscoring their adjuvant role. This comprehensive understanding of Trumenba’s structure–function relationship not only highlights the importance of lipidation in eliciting effective immune responses but also provides valuable insights for future vaccine development against NmB and other pathogens [42].

Therefore, to improve the efficacy of recombinant subunit vaccines, immunogens can be engineered as recombinant lipoproteins by adding lipobox DNA sequences into the N-terminus of the antigen-coding gene. Recent research has shown that lipoprotein SPs (SPs) can be effective vaccine adjuvants because they are recognized by toll-like receptor 2 (TLR2). For example, when an SP from *N. meningitidis* was linked to the dengue virus E3 protein, it created a lipidated version that significantly boosted antibody responses in mice. A similar strategy was employed with other bacterial, viral, and cancer antigens. Such recent advances highlight the lipoprotein SP role in vaccine technology [17,43,44,45].

In the present review, the aim is to provide a comprehensive examination of the biogenesis and immunological functions of bacterial lipoproteins. These proteins, characterized by their lipid modifications, play crucial roles in bacterial physiology and host interactions. Additionally, we will delve into the recent studies that investigate the utilization of lipo-box signal peptides (SPs) for the lipidation of heterologous proteins. This process not only enhances the stability and functionality of the proteins but also allows them to mimic naturally occurring bacterial lipoproteins. By highlighting these innovative approaches, we seek to underscore the potential of lipo-box-driven TLR2 activation in advancing modern vaccine design, thereby paving the way for more effective immunological applications.

## 3. Bacterial SPs Enable Lipoprotein Anchoring to Cell Membranes

Bacterial lipoproteins begin their synthesis within the cytoplasm as a precursor equipped with SP. Later, they are translocated across the membrane through a series of maturations via lipid modifications and SP cleavage [46]. The SPs of proteins in the periplasm and outer membrane are characterized by the N-terminal positively charged amino acids and a central hydrophobic region with uncharged amino acids. Moreover, SPs have a conserved lipo-box sequence at their C terminal. The lipobox sequence is [LVI][ASTVI][GAS][C] where cysteine residues are located at the N-terminus of mature lipoprotein and are the site for modifications with diacylglycerol and fatty acyl chains [47]. The SP is responsible for the translocation and directs the precursor to the Sec translocon, facilitating the passage of the nascent polypeptide chain into the membrane [48].

**Figure 2:**
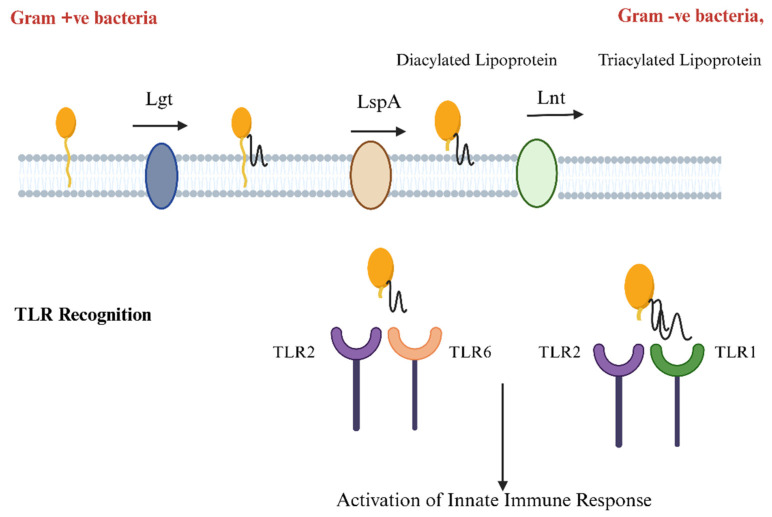
Lipidation and immune recognition: the role of Lgt, LspA, and Lnt in protein modification. Lipidation begins with Lgt adding lipids, resulting in diacylation in Gram-positive and triacylation in Gram-negative bacteria. LspA cleaves the signal peptide, and Lnt adds more lipids. These lipidated proteins activate immune signaling by engaging TLR2 with TLR1 or TLR6 (created with BioRender.com).

Once the pre-lipoprotein precursor is translocated across the membrane, it goes through a series of maturation steps, involving lipid modifications. The enzyme diacylglycerol transferase (Lgt) catalyzes the attachment of the diacylglycerol moiety via thioether linkage to the sulfhydryl group of cysteine residue of SP [49]. Following lipidation, lipoprotein signal-peptidase LspA (signal peptidase II) cleaves the SP and generates diacylglycerol pro-lipoprotein. On the cleavage of SP by LspA, the maturation of lipoprotein terminates in most of the Gram-positive bacteria [50]. On the other hand, Gram-negative bacteria undergo further lipidation by an enzyme called lipoprotein N-acyl transferase (Lnt) [51]. Lnt is responsible for the transfer of another acyl group from phospholipids into the cysteine residue, resulting in a mature triacylated lipoprotein. The triacylation improves the stability and integration of lipoprotein into the membrane [52,53]. Figure 2 explains the role of various enzymes in protein lipidation.

Upon maturation, the lipoproteins are localized either into the periplasm or inner membrane or sorted on the outer membrane. The lipoprotein outer membrane localization (LOL) pathway is the key mechanism for selectively transporting lipoproteins. The LolCDE complex, an ABC transporter located in the inner membrane, releases outer membrane specific lipoproteins upon ATP hydrolysis [54]. The lipoproteins destined for the inner membrane are not recognized by the LolCDE complex as they contain Asp at position +2, and thus they are not released [55]. Those lacking this sequence, (Asp +2), are released into the periplasm and bound by the LolA chaperone, which stabilizes the lipoprotein, shielding its hydrophobic region and prevents mislocalization into the inner membrane [56]. The lipoprotein-LolA complex further interacts with the LolB, an outer membrane specific receptor, which ensures the anchoring of the lipidated N-terminus with the lipid bilayer of the outer membrane. This step safeguards the precise sorting of lipoproteins on the outer membrane [57].

## 4. Decoding Bacterial Lipoproteins: How TLR2 Recognition Drives Immune Defense

**Figure 3:**
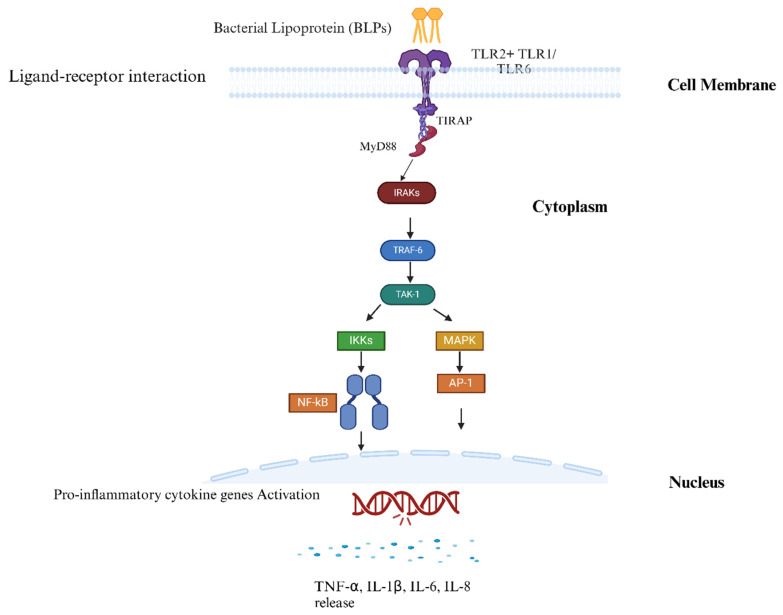
Activation of TLR2 signaling pathway by bacterial lipoproteins (BLPs). The TLR2 signaling pathway is triggered by TLR2 forming heterodimers with TLR1 or TLR6 upon binding bacterial lipoproteins, recruiting adaptor proteins like MyD88 and TIRAP. This activates IRAKs and downstream NF-κB, driving pro-inflammatory cytokine production (created in https://BioRender.com—accessed on 15 November 2024).

Bacterial lipoproteins are primarily recognized by toll-like receptor 2 (TLR2), which is facilitated by the lipid moieties. Further studies have disclosed that TLR2 can recognize the bacterial lipoproteins that are either diacylated or triacylated by forming heterodimers with TLR6 or TLR1, respectively. Kang and colleagues describe the crystal structure of the TLR2-TLR6 complex with diacylated lipopeptides, complementing earlier findings on the TLR2-TLR1 complex with triacylated lipopeptides and offering new insights into how TLR2 recognizes lipoproteins. TLR2 can heterodimerize with either TLR1 or TLR6, creating unique binding pockets that enable it to specifically recognize diacylated and triacylated lipopeptides [58]. This recognition is primarily driven by the leucine-rich repeat (LRR) domains of TLR2, where hydrophobic interactions between the ester-bound fatty acids of the ligand and the LRR9-12 modules of TLR2 play a crucial role [59]. At least two acyl chains with a minimum of 12 carbons each (eight in the case of murine TLR2) are required for effective TLR2 recognition. On the other hand, TLR2-TLR1 binding to triacylated lipopeptides involves an amide-linked fatty acid and a hydrophobic channel in TLR1; TLR6 has phenylalanine residues (F343 and F365) that block this channel. However, replacing these residues with methionine and lysine as in TLR1 allows TLR6 to form a complex with TLR2 and recognize triacylated lipopeptides. Unlike the TLR2-TLR1 heterodimer, where fatty acids bind separately to each receptor, the TLR2-TLR6 complex relies on hydrogen bonding between glycerol in the lipopeptide and the TLR LRR11 loops, stabilizing the dimer’s hydrophobic interface and ensuring specific TLR2-TLR6 binding through both the fatty acids and the peptide head group [60]. Figure 3 describes the immune recognition of bacterial lipoproteins by TLR2.

Recently, a study compared the innate immune response generated by diacylated and triacylated proteins. *Haemophilus influenzae* OMP26 was lipidated using SP which adds two fatty acids to mature protein. To further increase acylation, OMP26 was expressed in apolipoprotein N-acetyltransferase enzyme (Lnt)-rich *E. coli* strain, which successfully generated triacylated L-OMP26. Immune evaluations revealed that both di- and triacylation generated a significantly higher adaptive immune response as compared to non-lipidated OMP26 mice. However, the diacylated OMP26 response was comparatively higher than tri acylation [21,61].

Once dimerization is triggered by diacylated, triacylated lipoproteins or synthetic Pam2CSK4 or Pam3CSK4, it results in the activation of a signaling cascade that initiates with toll-interleukin-1 receptor domain-containing adaptor protein (TIRAP) binding to the TLR2. Later, the myeloid differentiation primary response 88 (MyD88) dependent pathway is triggered and causes the phosphorylation of interleukin-1 receptor-associated kinase 4 (IRAK4), IRAK1, and IRAK2. Further, it leads to the stimulation of TNF receptor-associated factor 6 (TRAF6), ultimately leading to the activation of nuclear factor kappa-light-chain-enhancer of activated B cells (NF-κB) and mitogen-activated protein kinases (MAPKs). Upon translocation to the nucleus, these transcription factors induce the production of pro-inflammatory cytokines (TNF-α, IL-1β, and IL-6), chemokines, and other mediators of innate immune response. TLR2 activation also stimulates the production of antimicrobial peptides and enzymes that directly attack the pathogen [62].

Aside from the immediate innate immune response, the TLR2 detection of bacterial lipoproteins helps to bridge the innate and adaptive immune responses. The pro-inflammatory milieu established by TLR2 activation is an important signal for the activation and maturation of dendritic cells (DCs), which are required to initiate adaptive immunity. Mature DCs increase the production of major histocompatibility complex (MHC) molecules as well as co-stimulatory molecules like CD80 and CD86, all of which are required for the efficient antigen presentation to T lymphocytes [63]. The interaction between DCs and T cells is critical for adaptive immune activation. Bacterial lipoproteins stimulate DC maturation, which improves their ability to prime naïve T cells and differentiates them into effector T cells. This mechanism is essential for the development of pathogen-specific CD4+ T helper (Th) cells, which can then differentiate into Th1, Th2, or Th17 subsets depending on the cytokine milieu. Th1 cells produce IFN-γ, activating macrophages to destroy intracellular pathogens [64].

Bacterial lipoproteins not only activate T cells but also alter B-cell responses. Cytokines released in response to TLR2 activation, particularly IL-6, promote the development of B cells into plasma cells capable of producing pathogen-specific antibodies. These antibodies serve an important role in neutralizing bacterial toxins, opsonizing bacteria for phagocytosis, and activating the complement system to lyse bacteria. The capacity of bacterial lipoproteins to elicit both T- and B-cell responses highlights their potential as vaccine antigens [65,66].

Compared to other TLR agonists, such as flagellin (TLR5 agonist) and heat shock protein 70 (Hsp70), the lipoprotein (Ag473) induces a more robust immune response. Infectious bursal disease virus (IBDV) antigen VP2 was formulated with Ag473 D1, flagellin, and Hsp70. When co-administered with VP2 or genetically linked, Ag473 produced significantly higher levels of cytokines IL-4, IL-12, and IFN-γ, as well as enhanced antigen-specific humoral and cellular immune responses. Notably, the immune response was more pronounced when Ag473 was genetically linked to VP2 than with co-administration. In this study, the importance is underscored of a strategy for converting non-lipidated antigens into potent lipidated antigens to elicit a robust immune response [67].

## 5. The Role of SPs in Vaccine Development, Diagnostics, and Therapeutics

Secretory proteins are produced with the SP (SP) located at the N-terminus, which directs the protein to its target location. Advances in research have unfolded various functional roles of SPs, which directly influence the characteristics of the associated “passenger” protein. Due to their role as a guiding sequence, SPs are used in diagnostics, vaccine development, and protein production platforms. Recent structural and computational tools have highlighted distinct regions within SPs and their specific functions, which together determine the fate of the passenger protein [47].

SPs are vital in recombinant protein production, especially for vaccine development. They facilitate the translocation as well as the expression of the recombinant proteins, assuring proper translation and localization. These peptides drive nascent proteins to translocation routes, the secretory (Sec) and twin-arginine translocation (TAT) pathways [68]. The Sec pathway is employed by both eukaryotes as well as prokaryotes to translocate unfolded proteins into the Sec translocon complex, making it suitable for vaccine proteins that require post-translational modifications [69]. However, the TAT pathway allows the translocation of fully folded proteins that require further structural integrity [70]. Beyond translocation, SPs also serve as allosteric activators of translocase. Once docked on SecA, SPs sequentially initiate the following three stages: a ‘triggering’ phase that lowers the activation energy, a ‘trapping’ phase engaging mature preprotein domains, and a ‘secretion’ phase that translocates the domains [71]. SPs also help in the identification of genes that encode for membrane-associated or secretory proteins by a signal-exon trap (SET) approach. SET benefits from the fact that SPs, located in the five prime-terminal exons, are characterized by a hydrophobic region that directs the proteins in secretion pathways or embeds them into the membrane [72]. By utilizing SPs to accomplish targeted section and proper folding, antigens that mimic natural confirmation and enhanced immunogenicity can be produced. For instance, N-terminal mu-phosphatase SP assisted in the production of glycosylated proteins in the COVID-19 vaccine [73].

SPs are regarded as multi-epitope domains with unique antigenic characteristics, illustrating their function beyond protein secretion. SP domains have a high density of T-cell and B-cell epitopes. SP-derived MHC-1 class, MHC class 2, and HLA-E epitopes were isolated and identified as potential vaccine candidates, which can activate CD4+ and CD8+ T-cell as well as B-cell responses. This SP-dependent approach is non-toxic, irrespective of HLA repertoire and valuable for patients with transporter-associated antigen processing (TAP) abnormality [74].

SPs have a variety of diagnostic and clinical applications. The role of SPs as diagnostic biomarkers has been unlocked. For instance, the role of SP was illustrated in pulmonary embolism as the level of SP complement for the epidermal growth factor domain-containing protein-1 indicates pulmonary embolism [75]. In gene therapy, SP is critical for directing fibroblast growth factors (FGFs) to secretory pathways, thus improving their targeted delivery in myocardial tissues. This targeted secretion aided by SP enhances regional blood flow more effectively than variants without SP. This testifies to the therapeutic application of SPs as they lower the required viral dose for gene therapy and improve clinical safety [76]. In another study, nuclear localization SPs play an effective role in the transport of genes into the nucleus and significantly enhance the gene expression levels in non-viral delivery systems [77].

Studies have suggested that mutations in SPs can lead to numerous diseases. Hamdi and colleagues concluded that 26 human diseases are associated with the SP mutations in 21 human proteins [78]. Mutations in pre-proinsulin SP were attributed to the onset of diabetes [79]. SP mutation also causes the autosomal-associated familial-isolated hypoparathyroidism [80]. A novel SP variation in the human luteinizing hormone receptor (LHCGR) influences receptor synthesis causing Leydig cell hypoplasia [81].

## 6. Bacterial SP Types: Structural Variations and Cleavage Pathways in Protein Targeting

SPs can be classified based on their structure as well as the cleavage mechanism of the signal peptidase. Archetypical SPs are characterized by a common tripartite structure, a positively charged N-terminal, a central hydrophobic region, and a C-terminal. The C-terminal contains a signal peptidase-specific sequence [82]. Lipoprotein SPs stand out by the presence of conserved lipo-box motif at C-terminal, critical for lipidation at cysteine residue, anchoring membrane proteins and cleavage by signal peptidase II [83]. Prepilin SPs, which interact with type IV pili assembly, have a basic domain at the C-terminus and lack substantial hydrophobic sections; they undergo breakdown by Type IV signal peptidases (SPase IV) [84]. In contrast, leader SPs lack large hydrophobic regions and are frequently present in proteins that must be secreted without membrane integration [85]. These SPs are cleaved by a variety of SPases. Most secretory proteins’ SPs are cleaved by type I SPase via a serine/lysine catalytic dyad mechanism [86]; type II SPase targets lipoprotein SPs following lipid modification [87]; and type IV SPase processes prepilin-like proteins implicated in type II secretion systems [88].

### 6.1. The Role of Bioinformatics: Tools for SP Prediction and Cleavage Site Detection

**Figure 4:**
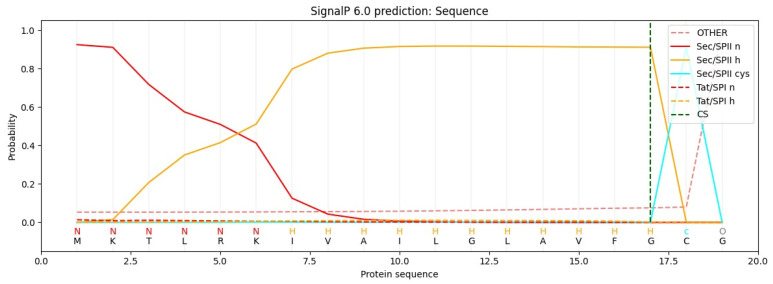
Signal sequence prediction by SignalP-6.0. The signal sequence was analyzed using bioinformatics, distinctly identifying the n, h, and c regions, along with precise marking of the cleavage site and lipobox (Created with https://services.healthtech.dtu.dk/services/SignalP-6.0/, accessed on 10 November 2024).

Bioinformatics tools play a crucial role in biomolecule screening [89]. SignalP-6.0 (https://services.healthtech.dtu.dk/services/SignalP-6.0/, accessed on 10 November 2024) is a tool for predicting SPs and their cleavage sites across diverse organisms. It differentiates between the following five types of SPs: Sec/SPI, Sec/SPII, Tat/SPI, Tat/SPII, and Sec/SPIII. SignalP-6.0 also identifies structural regions within SPs, such as the n, h, and c regions, as well as other SP-specific features [89,90]. For lipoprotein SPs, SignalP-6.0 can detect Sec/SPII, which are cleaved by signal peptidase II (Lsp) (Figure 4). Another tool, LipoP-1.0 (https://services.healthtech.dtu.dk/services/LipoP-1.0/, accessed on 10 November 2024), focuses specifically on lipoprotein SPs in Gram-negative and Gram-positive bacteria [91]. LipoP-1.0 is optimized for identifying signal peptidase cleavage sites I and II.

### 6.2. Using SPs for Lipidation of Other Proteins: Requirements and Validation

**Figure 5:**
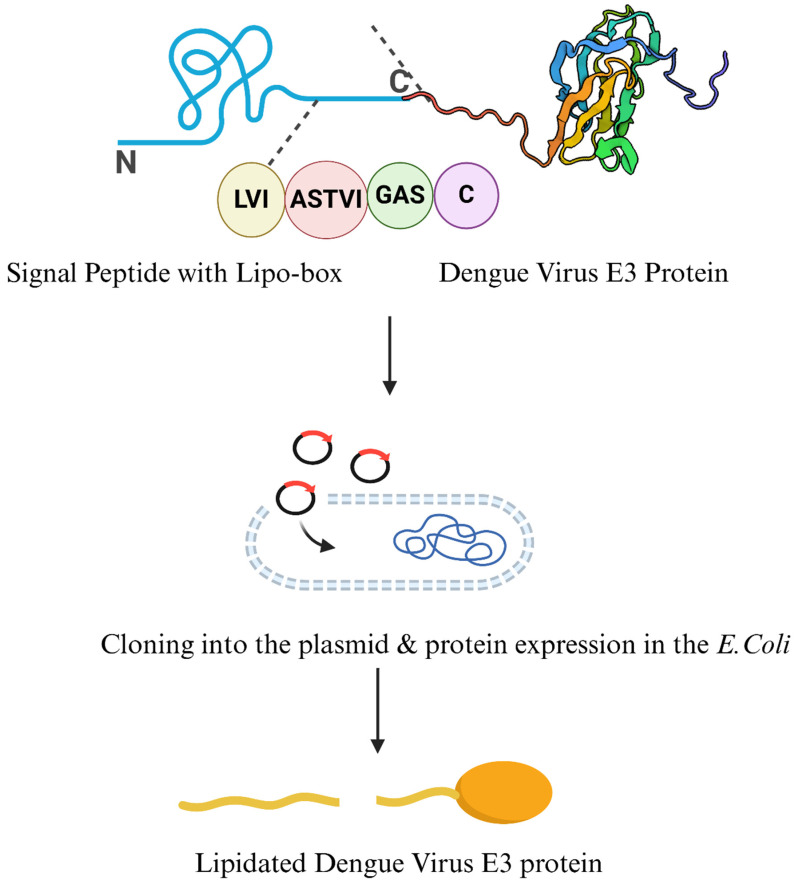
Lipidation of proteins: the critical role of the signal peptide lipobox. Signal peptide contains a lipobox which consists of a conserved cysteine residue at the +1 position, with [LVI][ASTVI][GAS]. This structural motif is essential for the attachment of lipid moieties. The E3 protein from the dengue virus has been cloned and expressed in an *E. coli* with SP lipobox, resulting in a lipidated form of the protein (Created in https://BioRender.com—accessed on 15 November 2024).

Bacterial lipoproteins, such as Ag473 from *N. meningitidis* and lipoprotein E from *Pasteurella multocida*, contain leader sequence SP featuring a “lipobox” motif, which is crucial for lipidation. During protein synthesis, this SP directs the protein to the bacterial membrane, where the peptide is cleaved off, leaving a cysteine residue at the N-terminus of the mature protein. This cysteine becomes the site of lipid attachment, anchoring the protein to the membrane and enhancing its ability to activate immune receptors such as toll-like receptor 2 (TLR2). This lipidation process not only stabilizes the protein but also enhances its immunostimulatory potential, making it a valuable feature for vaccine development. By applying similar bacterial lipoprotein SPs to heterologous proteins from other pathogens, such as the E3 protein of dengue virus, researchers can induce lipidation and improve immunogenicity, offering a promising approach to designing potent subunit vaccines and immunotherapies [17,43]. Figure 5 is the graphical illustration of this lipidation strategy.

Ag473 is a lipoprotein derived from *N. meningitidis*, comprising an N-terminal SP (SP) followed by three distinct domains, designated D1, D2, and D3. This lipoprotein is amenable to expression in *E. coli* C43(DE3) cells. To assess the potential application of Ag473 SP for lipidation of heterologous proteins, the dengue virus E3 protein was fused to Ag473 SP in multiple configurations, including SP alone, SPD1, SPD1D2, and SPD1D2D3. Notably, protein expression was absent when E3 was fused to SP alone, whereas IPTG induction was successful in the constructs containing SPD1, SPD1D2, and SPD1D2D3. These findings underscore the necessity of including specific residues downstream of the lipobox cysteine to facilitate protein induction [80,85].

Subsequent studies have demonstrated that lipidation of certain proteins, such as *H. influenzae* OMP26 and P6, can be achieved by appending the N-terminal lipoprotein SP sequences that incorporate a conserved lipobox motif (MKTTLKMTALAALSAFVLAGC). No additional sequences after the lipobox motif were added from native protein. Lipidation of the OMP26 and P6 fusion constructs was validated through activation of TLR2 reporter cells [46].

An alternative approach to lipidate heterologous proteins involved the design of the vector pETLip3, which integrates the endogenous signal sequence of *B. burgdorferi* outer surface protein A (ospA). Cloning and expression of target genes in pETLip3 led to lipoprotein production, as illustrated by the successful lipidation of *S. pneumoniae* proteins DacB and PnrA. This lipidation was confirmed by liquid chromatography–mass spectrometry (LC-MS). Immunization with these lipidated antigens resulted in reduced pneumococcal colonization and promoted a Th1-skewed immune response [23].

In conclusion, the addition of an SP containing a lipobox sequence presents a viable strategy for lipidating heterologous proteins. However, the choice of SP and fusion methodology should be tailored to the specific requirements and objectives of the experimental design.

### 6.3. Lipidation by SPs: A Multifaceted Approach to Enhancing Vaccine Efficacy Across Viral, Bacterial Pathogens, and Cancer Antigens

Lipoprotein SPs have recently been explored as vaccine adjuvants. In one study, an SP sequence from *N. meningitidis* (Ag473) was genetically linked to the E3 protein antigen from the dengue virus. As expected, the SP cleaved off, resulting in lipidated E3, which was then used to immunize mice. Lipidation of E3 was confirmed through Q-TOF mass spectrometry, and mice in the lipidated E3 group showed significantly higher IgG and virus-neutralizing antibody titers than those given non-lipidated E3 [92]. Additionally, gene deletion studies later demonstrated that rlipo-D1E3 lipoprotein and synthetic lipopeptide Pam3 activated TLR2-dependent NF-κB signaling and cytokine release. The absence of any immune response in TLR2^−/−^ mice further emphasized the specificity of TLR2-mediated signaling, while wild-type and TLR4^−/−^ mice responded effectively to the lipoproteins. Cytokines such as p38, IL-23, and ERK1/2 were significantly higher in the lipidated E3 group compared to Pam3, highlighting the enhanced immunogenicity of lipidated antigens [93].

Considering these findings, lipidation has been applied to antigens from other pathogens to enhance immunogenicity. *Clostridium difficile* infections (CDI), a significant threat in hospital settings, are driven by the exotoxins TcdA and TcdB. The receptor-binding domains (RBD) of TcdA showed promise in protecting against CDI, with lipidation of these RBDs (via the incorporation of a lipobox) yielding a 10-fold increase in potency over non-lipidated RBDs, offering 90–100% protection against lethal CDI [18]. A similar approach was applied to combat *S. aureus* infection, targeting the immune-evasive formyl peptide receptor-like 1 inhibitor protein (FLIPr). Lipidation of FLIPr via Ag473 SP boosted both mucosal and systemic immunity, effectively blocking FLIPr-mediated phagocytosis. Thus, lipidation robustly enhances immune responses across various immune pathways [19].

Against the Zika virus, the envelope protein domain III (rZE3) was lipidated using the same strategy by Ag473. rLZE3 was successfully lipidated to activated dendritic cells (DCs), and later taken up by dendritic cells. Immunization of C57BL/6 mice with rLZE3 revealed higher neutralizing antibody titers with prolonged protection after Zika virus challenge compared to non-lipidated rZE3 [20].

In the case of respiratory infections caused by *H. influenzae*, which result in high morbidity and mortality, there is a pressing need for effective vaccines. The potent antigens P6 and OMP26 have shown potential as subunit vaccine candidates. When these antigens were lipidated with an SP (MKTTLKMTALAALSAFVLAGC), they induced significantly higher antibody titers and cytokine responses in mice, providing robust protection against middle ear infections by non-typeable *H. influenzae* (NTHi). Additionally, lipidated antigens reduced NTHi colonization in the nasopharyngeal surfaces, further highlighting the potential of lipidation in enhancing vaccine efficacy [21].

The lipidation studies have increased their horizons and participation in cancer studies. *Human Papillomavirus* (HPV) antigen E7 was formulated with a lipoprotein derived from Ag473 using tryptic digestion. The vaccine complex led to a sharp increase in anti-E7 antibody titers with Th1-biased cytokine release. In a TC-1 mice tumor model, rE7, the lipoprotein complex inhibited tumor growth while rE7 failed to do so alone [22]. The case studies are summarized in Table 1.

These studies collectively suggest that the lipidation of diverse antigens enhances immune responses, regardless of the antigen’s origin, whether bacterial-, viral-, or cancer-related. Lipidation has been shown to activate both innate and adaptive immune responses in various disease models, resulting in a significant increase in antibody and cytokine levels [94]. These findings underscore the potential of lipoprotein SP as an effective lipidation agent and TLR2 agonist in vaccine formulations.

### 6.4. Challenges and Solutions in Lipidated Protein Expression: Case Studies and E. coli Strain Optimization

One of the main challenges in scaling-up these vaccine formulations lies in the choice of host organism. Various *E. coli* strains have been tested for their ability to express lipidated proteins, with the JM109(DE3) strain failing to produce significant expression. The BL21(DE3) strain, though capable of higher expression levels, struggled to incorporate the necessary lipidation modifications. However, the C43(DE3) strain stood out as the most successful, as it was able to achieve both high levels of protein expression and proper lipidation, confirmed through mass spectrometry analysis. This finding highlights the importance of selecting the right bacterial strain for production, as not all strains can efficiently perform lipidation at industrial scales [95].

In addition to strain selection, several environmental factors significantly influence the lipidation process. Temperature, pH, and nutrient composition of the culture medium play key roles in the efficiency of protein lipidation. For example, studies on the lipidation of OMP26 proteins demonstrated that culturing *E. coli* C41(DE3) cells in M9 minimal medium at 30 °C overnight resulted in a higher yield of lipidated proteins. Moreover, maintaining a pH above 7 promoted the triacylation of proteins, as the activity of the Lnt enzyme—responsible for adding a third fatty acid to the protein—was enhanced at a basic pH. This highlights the need for the precise control of these parameters during large-scale production [21].

The expression of lipoproteins under standard conditions is often challenging and necessitates further optimization of the expression parameters to achieve successful production. Despite these advances, challenges remain in optimizing the expression and lipidation of lipoproteins under standard conditions. Scaling-up production requires the continuous refinement of expression parameters, such as optimizing temperature, pH, and media components, to achieve the desired protein yield and lipidation efficiency. Furthermore, the industrial production of SP-containing vaccines may require the development of specialized expression systems or the engineering of strains that can more effectively handle lipidation processes at larger scales. These challenges must be addressed to ensure the efficient, cost-effective production of lipidated protein vaccines, particularly for use in global vaccination campaigns [96].

## 7. Conclusions

In conclusion, in this review, the crucial role is highlighted of bacterial lipoprotein SPs in driving vaccine advancements, particularly through their ability to engage immune pathways like TLR2 signaling. The successful use of native bacterial lipoproteins in vaccines for diseases like meningococcal and Lyme illustrates their strong potential for triggering immunity through TLR2 activation. Notably, lipoprotein SPs could be instrumental not only in bacterial vaccines but also in strategies targeting viral and cancer antigens, showing a broad potential. Moving forward, the research should prioritize refining lipidation processes via SPs and improving expression systems to enhance lipoprotein production and effectiveness. Exploring the structural diversity and cleavage patterns of these peptides could lead to more stable, immune-active vaccine designs. With their promise as foundational elements in next-generation vaccines, lipoprotein SPs stand out as key tools. To fully unlock this potential, a combined approach involving bioinformatics, molecular biology, and immunology will be critical in developing new vaccines against emerging health threats.
